# Navigating the Hump: Managing Moynihan’s Hump During a Laparoscopic Cholecystectomy

**DOI:** 10.7759/cureus.94791

**Published:** 2025-10-17

**Authors:** Allison Meihofer, Kimberly Cortez Perez, Leandro Tapia Garcia, Necial Marcelin, Mohammad Masri

**Affiliations:** 1 General Surgery, Nova Southeastern University, Dr. Kiran C. Patel College of Osteopathic Medicine, Davie, USA; 2 Surgery, University of Medicine and Health Sciences, Basseterre, KNA; 3 Surgery, Universidad Nacional Pedro Henríquez Ureña, Santo Domingo, DOM; 4 General Surgery, Larkin Community Hospital, South Miami, USA

**Keywords:** biliary anatomic variations, caterpillar hump, complications, critical view of safety (cvs), moynihan’s hump

## Abstract

Moynihan’s hump is a rare variant of the right hepatic artery (RHA) characterized by a tortuous RHA coursing near the cystic duct. This complicates cystic artery (CA) identification, increasing the chances of mistaking the RHA for it, resulting in inadvertent injury during a cholecystectomy. We present the case of a Moynihan's hump identified during the critical view of safety assessment in a cholecystectomy performed on a 47-year-old male with cholelithiasis. Our report highlights the surgical challenges, emphasizing the importance of the identification of key structures and techniques to minimize complications. Flexibility in surgical planning and awareness of anatomical variations are crucial for ensuring patient safety.

## Introduction

Moynihan’s hump, or caterpillar hump, is a rare anatomical variation of the right hepatic artery (RHA) characterized by an unusual course that brings the artery into proximity with the cystic duct. This configuration creates significant challenges during cholecystectomy procedures, as the RHA may be mistaken for the cystic artery, leading to potential vascular injuries. The reported incidence of Moynihan’s hump varies between 1.3% and 13.3% of cases [1].

In standard biliary anatomy, the cystic artery typically branches from the RHA within Calot’s triangle to supply blood to the gallbladder. However, in the presence of Moynihan's hump, the RHA’s anomalous, looping course can result in a shortened or obscured cystic artery, complicating its differentiation during dissection. Failure to recognize this variation increases the risk of ligation or injury of the RHA, which can lead to severe complications such as hepatic ischemia, bile duct injury, and severe hemorrhage [2].

The etiology of Moynihan’s hump is not fully understood, but it is believed to result from variations in the embryological development of the hepatic arterial system, where abnormal branching or looping of the RHA occurs during vascular formation [3]. While traditional preoperative imaging studies, such as standard ultrasound and routine CT scans, often fail to reveal this anomaly, advanced imaging techniques like CT angiography or magnetic resonance angiography (MRA) have the potential to identify unusual arterial courses, including Moynihan’s hump [4]. However, these specialized studies are not commonly employed preoperatively unless there is a known risk or suspicion of vascular anomalies. Surgeons must be vigilant in identifying and preserving the RHA during cholecystectomy, especially when encountering unexpected anatomical configurations [3].

This presentation highlights the importance of recognizing vascular anomalies, emphasizing why surgeons must remain vigilant and adaptable during procedures. Understanding this anatomical variant is critical, as it underscores the necessity of the Critical View of Safety (CVS) protocol and other measures designed to minimize operative risks. Furthermore, it reinforces the value of surgical education in addressing variations to enhance patient safety and improve surgical outcomes.

## Case presentation

This case presents a 47-year-old male with symptomatic cholelithiasis, whose surgery was complex due to the unexpected presence of Moynihan's hump. The patient, with a past medical history of hypertension, gastroesophageal reflux disease (GERD), prior appendectomy, and former tobacco use, presented to Larkin Community Hospital (LCH) with a 3-day history of bilious vomiting, occurring approximately 20 times, accompanied by nausea. The patient also reported chronic right upper quadrant abdominal pain, which was exacerbated by the consumption of greasy or fatty foods, as well as one episode of subjective fever.

**Figure 1:**
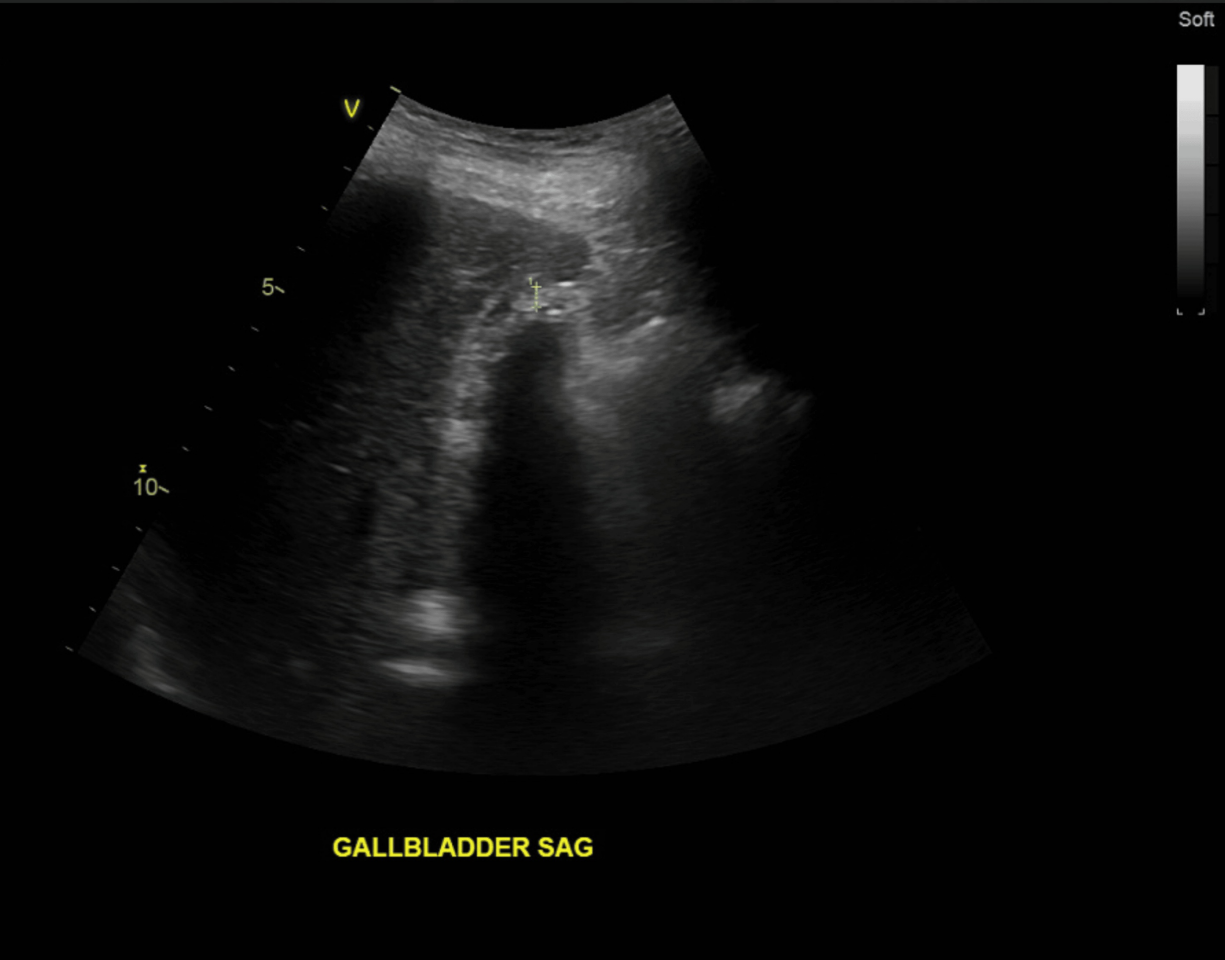
Ultrasound of the gallbladder showing a wall-echo-shadow sign Abdominal ultrasound, sagittal view of the gallbladder, obtained with a curvilinear transducer, demonstrating the wall-echo-shadow (WES) sign consistent with cholelithiasis.

**Figure 2:**
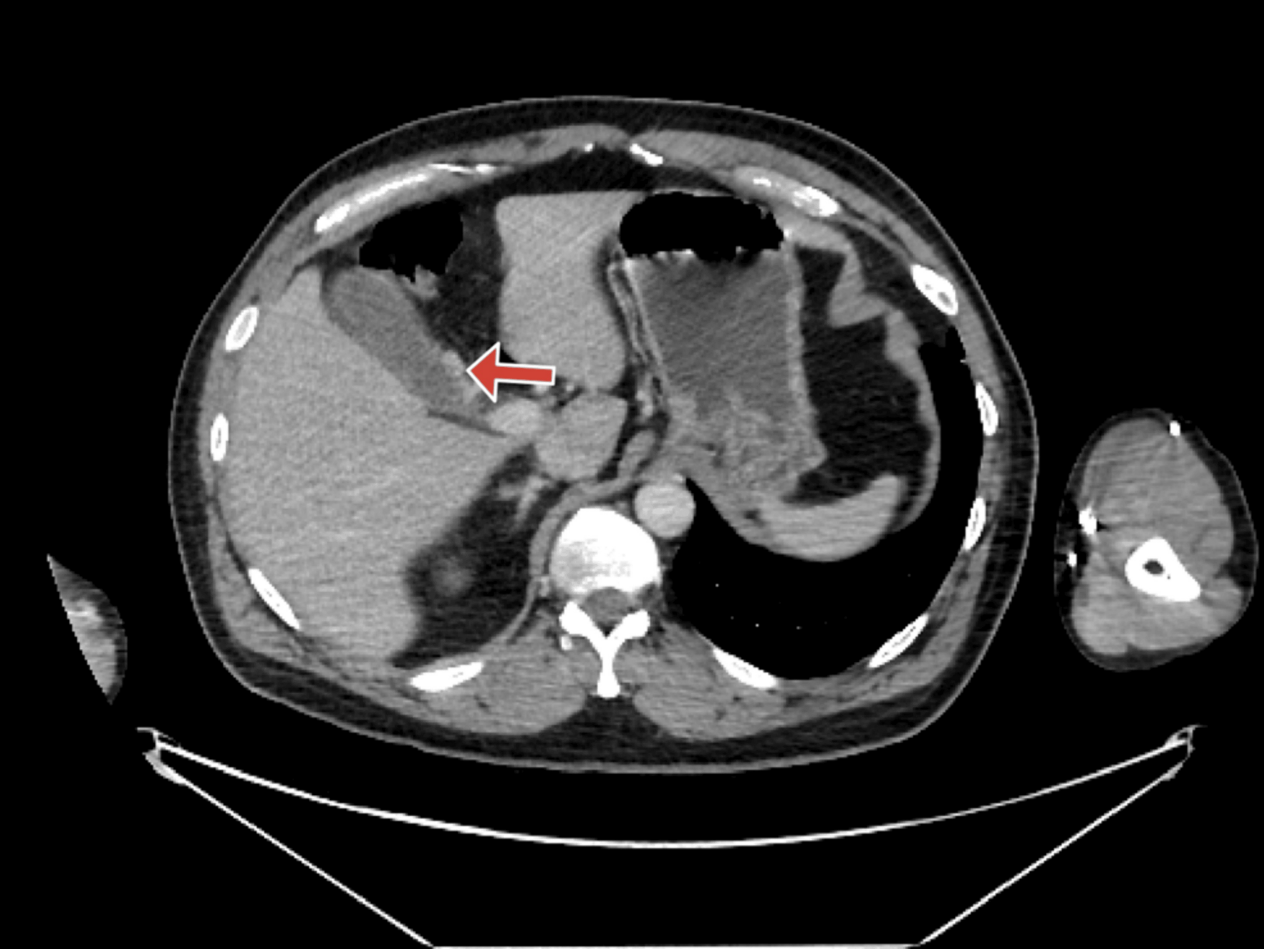
Computed tomography (CT) image of the cystic artery Contrast-enhanced abdominal CT, axial view in the portal venous phase, showing the cystic artery (arrow).

**Figure 3:**
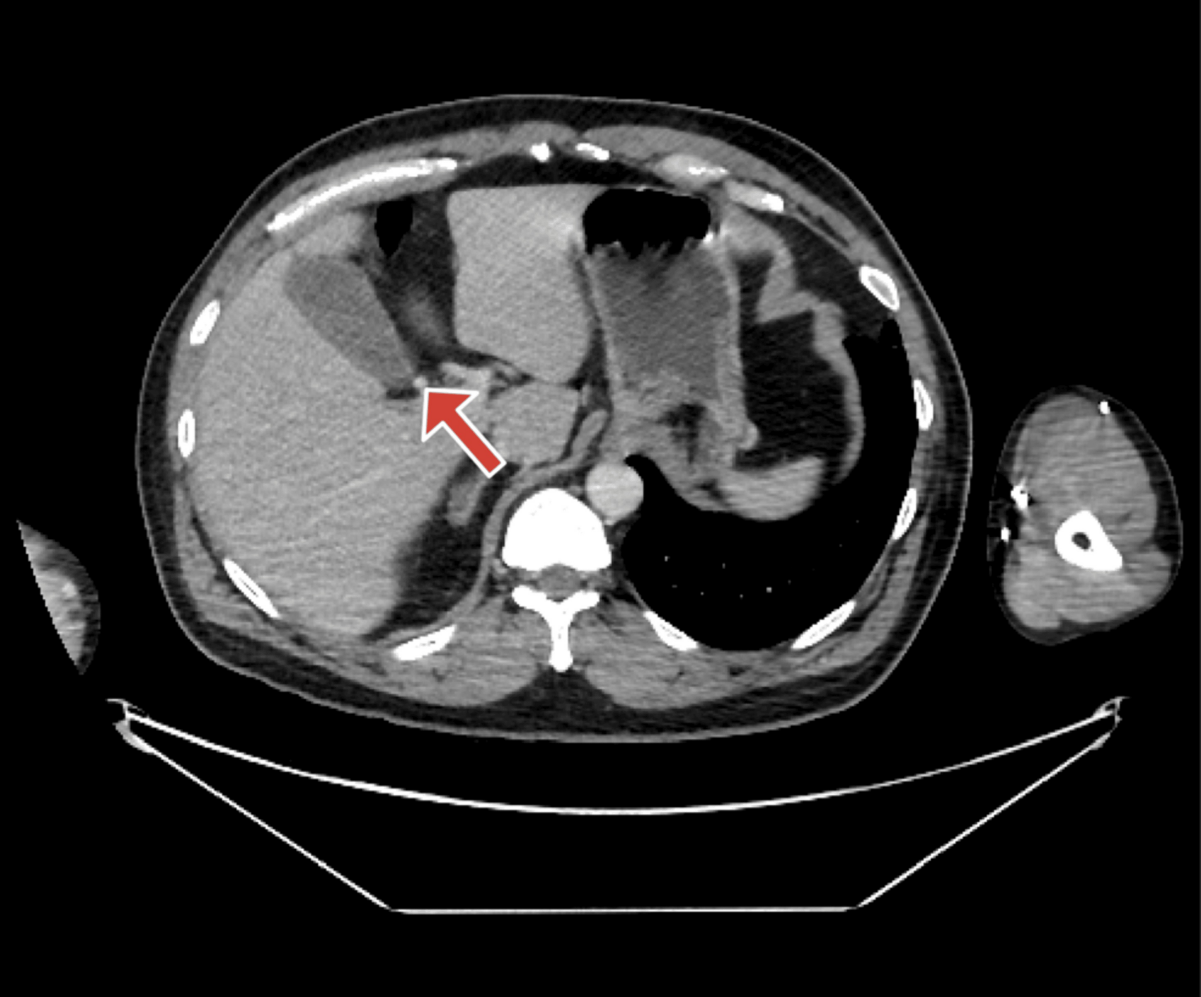
Computed tomography (CT) image of the right hepatic artery arching behind the common bile duct (CBD) Contrast-enhanced abdominal CT, axial view in the portal venous phase, obtained at a more superior level than Figure 2, demonstrating the right hepatic artery coursing posterior to the common bile duct and continuing distally (arrow).

On physical examination, the patient exhibited a positive Murphy's sign. Additionally, an abdominal ultrasound showed a wall echo shadow sign, suggesting the presence of cholelithiasis, and demonstrated thickening of the gallbladder wall, suggesting underlying inflammation (Figure 1). The CT showed the cystic artery seen coming off on axial (Figure 2) and more superior showing RHA arching behind the common bile duct (CBD) and continuing distally (Figure 3). Laboratory evaluations, including complete blood count (CBC), liver function tests (LFTs), and lipase, were within normal limits. The clinical presentation was consistent with biliary colic, and the patient was made NPO (nothing by mouth) and scheduled for laparoscopic cholecystectomy (LC).

**Figure 4:**
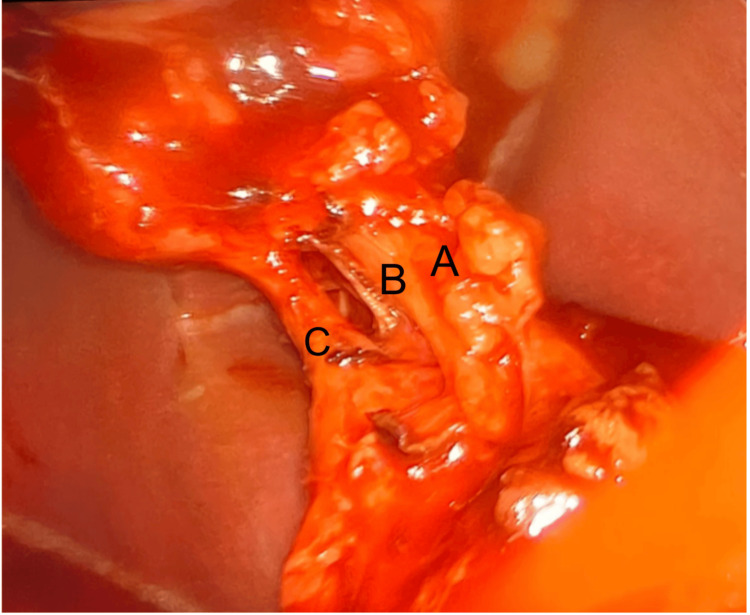
Intraoperative photograph demonstrating the right hepatic artery (A), cystic artery (B), and cystic duct (C)

**Figure 5:**
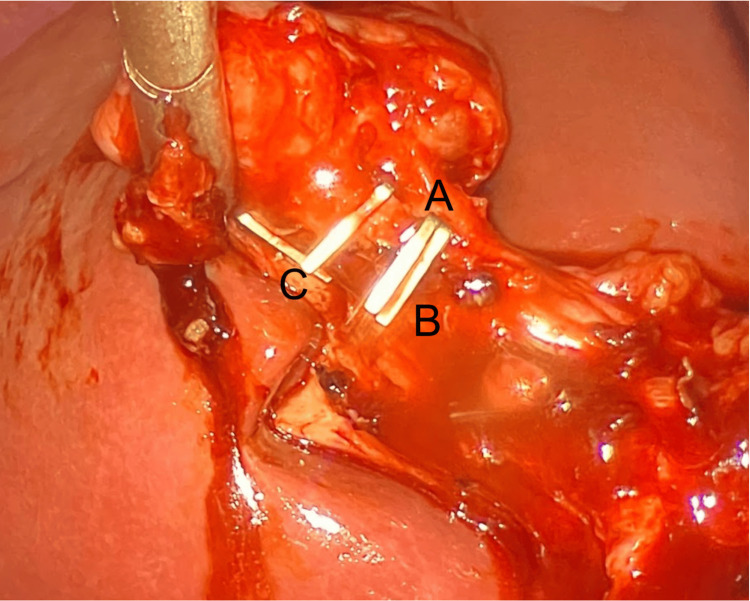
Intraoperative photograph following cystic duct clipping, showing the right hepatic artery (A), cystic artery (B), and clipped cystic duct (C)

During the laparoscopic procedure, Calot's triangle was carefully dissected to ensure all critical structures were clearly visualized. The dissection revealed a rare anatomical variant of the gallbladder anatomy, known as Moynihan’s hump or caterpillar hump, where the RHA courses close to the cystic duct (Figure 4). To prevent potential complications, such as vascular ligation or injury to the bile duct, careful dissection techniques were used to differentiate the RHA from the cystic duct and cystic artery. We could see the RHA, cystic artery, and clipped cystic duct (Figure 5). The gallbladder was successfully removed, keeping Moynihan's hump intact. The patient made a full recovery, with complete resolution of symptoms following the procedure.

This case highlights the importance of proper identification and careful surgical techniques when performing a laparoscopic cholecystectomy, especially in the presence of anatomical variations such as Moynihan’s hump.

## Discussion

When planning hepatobiliary surgery, it is important to consider the various anatomical variations in the biliary tree and hepatic vasculature, as they can lead to numerous challenges. The prompt identification of these variations is essential in preventing inadvertent injury to the vasculature during laparoscopic cholecystectomies. One of these variants, known as Moynihan’s or caterpillar hump, is defined by a tortuous RHA that loops near the cystic duct [5]. The incidence of this variation in the literature ranges from 1.3% to 13% of the population [1]. While rare, the presence of Moynihan’s hump can cause surgeons to mistake the RHA for the cystic artery (CA), resulting in serious complications such as hepatic ischemia, bile duct injury, and hemorrhage [1].

Moynihan’s hump can be found anterior or posterior to the common hepatic duct, although it is most commonly seen anteriorly, and may form either a single or double loop. The origin of the cystic artery relative to the hump can also vary. If the CA is proximal to the loop, it is elongated and crosses the RHA to reach the gallbladder. If the CA is found distal to the loop, which is more common, it is short [1]. In our case, Moynihan’s hump was a single loop found anterior to the common hepatic duct, with a short cystic artery arising distally to it. These anatomical differentiations make identifying and differentiating the cystic artery from the RHA more challenging during dissection.

Failing to recognize this variation during careful dissection in Calot's triangle can lead to several complications. If the RHA is mistaken for the CA, it can be mistakenly ligated or transected, leading to lobar hepatic ischemia or necrosis [2]. Partial ligation can result in the formation of a hepatic aneurysm, increasing the risk of hemorrhage. Additionally, excessive bleeding of the RHA during a procedure can obstruct the surgeon's view, increasing the chances of bile duct injury and possibly requiring an open cholecystectomy instead [2].

Given these risks, the proper identification of vascular structures in this area is crucial. To reduce these risks, surgeons use a technique known as the CVS, which involves three essential steps. First, the hepatocystic triangle, also known as Calot’s triangle, is carefully dissected, ensuring no tissue is blocking key structures. Next, the lower one-third of the gallbladder is separated from the liver to expose the cystic plate, confirming that the gallbladder is attached to the liver bed. Lastly, the surgeon should verify that only two structures are attached to the gallbladder: the cystic duct and the cystic artery [6]. Additionally, administering an IV bolus of indocyanine green dye can help visually enhance the cystic artery and hepatic artery, further aiding in safer dissection [7].

Studies suggest that the risk of injury during laparoscopic cholecystectomy complications increases when surgeons confuse anatomical variations with normal anatomy. To minimize these complications, every surgeon needs to be aware of these variations and prepared to adapt when encountering unusual structures such as Moynihan's hump [8]. This case highlights the importance of close attention to detail and adaptability during surgical procedures, especially when identifying unexpected vascular anomalies. Proper anatomical education, continuous skill refinement, and adherence to safety protocols like the CVS are crucial to reducing inadvertent injuries and improving patient outcomes. Surgeons must remain aware and prepared to ensure safety during all biliary procedures.

## Conclusions

Moynihan’s hump is a rare but important variation in biliary anatomy that presents unique challenges during laparoscopic cholecystectomy. Failure to identify this anomaly can lead to significant complications, including vascular injury, hepatic ischemia, and uncontrolled bleeding. This case highlights the importance of meticulous dissection, adherence to the CVS, and intraoperative vigilance when identifying and preserving key vascular structures during surgical procedures. It is vital for surgeons to understand biliary and hepatic arterial variations to minimize the risk of injury and improve patient outcomes. Overall, maintaining preparedness, following safety protocols, and having flexibility in surgical planning are essential to ensure safe and effective management of anatomical variations like Moynihan’s hump.
